# Home as Haven: Palliative and End‐of‐Life Care for People with Dementia in Non‐Residential Settings

**DOI:** 10.1002/alz70858_102811

**Published:** 2025-12-25

**Authors:** Dáithí Clayton

**Affiliations:** ^1^ DEEP Network, Aalter, Aalter, Belgium; Flemish Dementia Working Group, Antwerp, Belgium

## Abstract

As dementia progresses to advanced stages, the need for compassionate and person‐centred care becomes paramount. While institutional settings are often considered default options for palliative and end‐of‐life care, many individuals with dementia and their families express a strong preference for remaining at home. This presentation will explore the unique challenges and opportunities of providing high‐quality palliative care for people with dementia in non‐residential environments, drawing from the experience of Mens & Zorg, an award‐winning Flemish healthcare company specializing in at‐home dementia care. Key themes will include fostering trust with families, addressing complex medical and psychosocial needs, and developing tailored approaches that respect the dignity, autonomy, and preferences of individuals with dementia. The presentation will also highlight strategies for interdisciplinary collaboration and integrating community resources to create a network of support that extends beyond the home. In addition, this session will explore the innovative use of technology and adaptive tools to support caregivers and enhance quality of life during end‐of‐life stages. Through case studies and qualitative data, the presentation aims to provide practical insights into how person‐centred palliative care can empower people with dementia to experience comfort and continuity in the familiarity of their own homes.

**Key Takeaways**: Strategies for delivering holistic, person‐centred palliative care in home settings. Tools and resources to support caregivers in managing complex end‐of‐life needs. Insights from Flemish healthcare models that emphasize dignity, respect, and autonomy. This presentation aims to inspire healthcare professionals, policymakers, and researchers to prioritize home‐based palliative care as a viable and humane option for people with dementia.

